# Soil Recycling Geopolymers Fabricated from High Power Ultrasound Treated Soil Slurry in the Presence of Ammonia

**DOI:** 10.3390/ma12223804

**Published:** 2019-11-19

**Authors:** Louis-Marly Kwedi-Nsah, Yuta Watanabe, Takaomi Kobayashi

**Affiliations:** 1 Department of Energy and Environment, Nagaoka University of Technology, 1603-1 Kamitomioka, Nagaoka, Niigata 940-2188, Japan; Knsah@yahoo.com; 2 Department of Science and Technological Innovation, Nagaoka University of Technology, 1603-1 Kamitomioka, Nagaoka, Niigata 940-2188, Japan; Yuta_watanabe@stn.nagaokaut.ac.jp

**Keywords:** organic carbon, soil reuse, soil treatment, high-power ultrasound, geopolymer, soil washing

## Abstract

Soil slurry was recycled to prepare a geopolymer after treatment with high-power ultrasound (US) in the presence of NH_3_, HCl, and NaOH. Under 28 kHz US, 0.1 M NH_3_ additives effectively decarbonized the slurry, eliminating 72.2% of the carbon content from the original soil. The US-treated soils were used as raw materials for the geopolymer, as they contained Si and Al components in the range of 25–30 and 8–10 wt.%, respectively. The geopolymer was prepared with a Na_2_SiO_4_/NaOH aqueous solution at a ¼ weight ratio at 80 °C for 24 h. The resultant geopolymers from the NH_3_-treated soil showed the best compressive strength of 3 MPa after 1 day of curing, with a low carbon content, when NH_3_ was used as an additive as opposed to HCl and NaOH under 1200 US exposure.

## 1. Introduction

A geopolymer is a ceramic material that can be prepared at low temperatures in the presence of an alkaline activator [1]. The fact that ceramic blocks can be prepared at low temperatures is an advantage over other materials and has drawn the attention of researchers focused on developing sustainable materials that can be of value in the reuse of fly ash and other inorganics [2]. Since the interest in sustainability has risen in the construction sector [3], geopolymer research has recently seen significant growth. The pioneering research on geopolymers shows highly promising results relating to durability and fire resistance [4,5].

It is interesting to note that soil contains minerals, water, air, gases, organic matter, and microorganisms [6,7,8]. The mineral part is the largest component, with sand, silt, and clay comprising approximately 45%–50% of the total soil. Thus, soil is typically made up of SiO_2_, Al_2_O_3_, Fe_2_O_3_, and CaO constituents. The organic matter in soil is mainly derived from dead plants and animals, while at low percentage concentrations, the carbon parts are decomposed by microorganisms [9]. Generally, when soil has to be recycled, it must be sintered at high temperatures to remove such organic components [10,11,12,13,14]. As organic carbon parts are known to decrease the strength and stability of inorganic ceramic materials, less carbon content in soil is, therefore, an important factor to consider during soil recycling for the application of geopolymers [15]. 

So far, the ideal method of eliminating soil carbon remains to be discovered. Currently, the most prominent method is shaking the soil in water for several hours [16]. Usually, water or aqueous chemicals, such as acids, bases, surfactants, and chelating agents, are vital for the conventional washing method [17]. This technology is recognized as an important method for the elimination of hazardous contaminants and for washing heavy-metal-contaminated soils [18]. However, the drawback of this soil washing technique (with the addition of reagents in sequential washing steps) [19] is that the washing process can remove pollutants or unwanted compounds only from the surface of the soil particle [20,21]. 

Ultrasound (US), which is the vibration of sound waves above an audible frequency, causes remarkable mechanical soil dispersion. This is because the bubbles generated by US inside the reaction vessel oscillate/increase in size and then explode with a violent power, generating extreme sonochemical and sonophysical effects in aqueous soil [22,23]. It is known that both effects are applied in some technologies for surface cleaning, extracting, and micromixing [24,25,26,27]. For US soil washing, in addition to other factors, the effect of sonication power has been reported in several papers [24,25,26]. Sonication power is known to accelerate sequential extraction by US washing. Also, US waves can break down the particle size of soil and aggressively agitate a slurry solution. 

Although excavated soil is used as a renewable material in the construction industry, the recycling rate, however, seems very low [28]. This is because these soils are usually categorized as waste [29]. From a financial perspective, soil displacement represents about 5%–16% of the capital cost of every project [30]. In South Korea [31,32], Canada [33], and Italy [34], for example, programs have been created to encourage the reuse of excavated soil. In Japan as well, due to the limitation of available natural resources and land space for landfills, reuse in construction fields is greatly encouraged [35]. Since these waste soils can be converted to other useful products, it is quite meaningful to study soil reuses. 

As previously mentioned, soil clay is generally rich in Al_2_O_3_ and SiO_2_ components, allowing the possibility for soil to be reused as inorganic materials. Although the research is limited, one of the major possibilities of soil reuse is for the fabrication of geopolymers. An attempt was made by Singhi et al. to investigate soil–geopolymer incorporated with slag, fly ash, and a mixture of slag and fly ash [36,37]. The addition of nanoparticles of layered mineral silicates led to the flocculation of clay particles, which improved the rheological properties of geopolymer nanocomposites, suitable to be used in 3D printing applications [38,39]. In the present study, high-power US soil washing is performed, and the treated soil is used as a geopolymer source material. 

## 2. Materials and Methods

**Figure 1:**
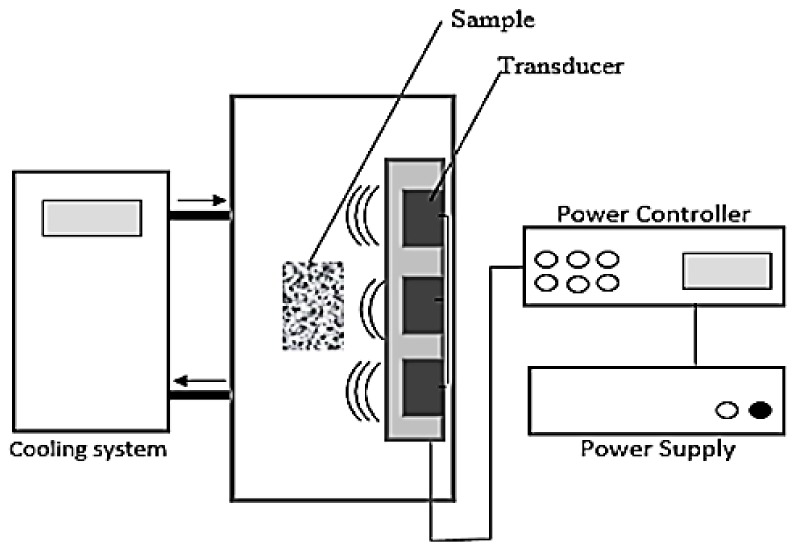
Upper view of ultrasound experimental setup.

Waste soil was sampled from Niigata (Japan) and it was used for this study without further treatment. The soil sample which was classified as sludge (ρ = 1.3 g/cm^3^) was air-dried, ground, and passed through a 1.18 mm sieve before treatment. The experiment was carried out in a stainless-steel bath (10 cm length × 60 cm width × 50 cm depth) with a submersible stainless-steel rectangular container housing seven transducers (42 × 30 × 10 cm^3^). The US was generated with a resonance frequency of 28 kHz by a Honda electronic device (Dynashock WD 1200-28, Toyohashi, Japan) and 300–1200 W output power. For US washing of the excavated soil, the ratio of soil and washing liquid was set at 1:10 and the sample vessel was placed 5 cm from the transducer heads. The upper view of the US setup used for treatment was as shown in Figure 1.

The slurry sample was exposed to different ultrasonic intensity for a period of 60 min. During this study, 0.01–1 M aqueous solutions of HCl (acid), NaOH (strong base) and NH_3_ (weak base) were prepared and used as treatment additives. At the end of each US treatment, the slurry mixture was filter-pressed (Nihon-RokaSochi, Nagoya, Japan) with filter cloth (0.3 cm^3^/cm^2^.sec) for effective separation of the solid and liquid content. For the determination of soil components, the soil extracts were dried at 40 °C for 24 h. The dried extracts were then fused in pellets and the mineral content of the soil was analyzed by XRF (Rigaku ZSX Primu II, Rigaku Corp., Tokyo, Japan), Table 1 shows the effect of US on the soil chemical constituent when different additives were used for soil washing.

To evaluate the percentage efficiency of US to Carbon in soil (CIS) extraction, the following formula was used:

(1) $$\mathrm{CIS}=\frac{XRF\;value\;before\;US-XRF\;value\;after\;US}{XRF\;value\;before\;US}\;\times 100.$$

**Figure 2:**
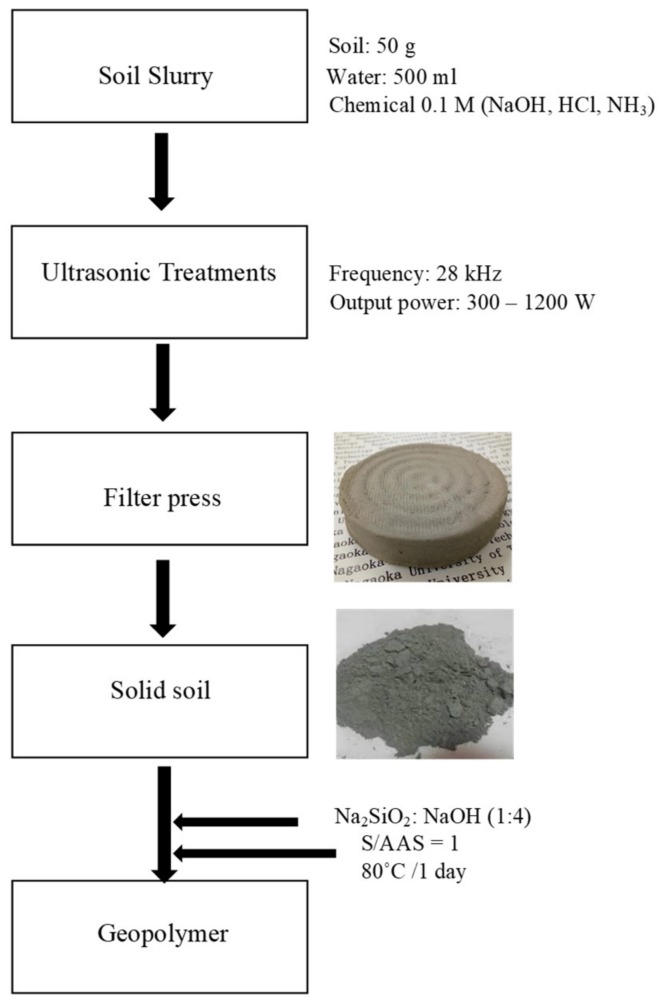
Protocol for US soil washing, and geopolymer fabrication

After XRF analyses, the remaining solid extract was crushed and passed through a 0.25 mm sieve and fine soil powder was obtained. Considering the numerous generic information available on geopolymers, a rigorous trial-and-error method was adopted to fabricate soil-based geopolymer concrete similar to the technology currently used to manufacture fly ash-based geopolymer concrete. The fabrication of soil geopolymer was carried out step by step as shown in Figure 2.

An alkalis activator was prepared using 1 M aqueous Na_2_SiO_3_ with 10 M NaOH at a ratio of 1:4, meanwhile, the weight ratio of soil (S) and the alkali activator solution (AAS) was fixed at 1:1. The US treated soil powder was mixed with the AAS and then stirred for 60 s forming a paste. The paste was poured into a plastic mold (30 × 30 × 30 mm^3^) and cured in an oven (Avo 200NS, ASONE-Japan, Osaka, Japan) at 80 °C for 24 hr. After curing, the samples were crushed for further characterization. The X-ray Diffractometer (XRD) (Rigaku smart lab 3 kW, Tokyo, Japan) was used with Cu Kα radiation at 40 kV and 30 mA. The sample was measured on a glass plate after crushing, with scanning angle 10–60 degrees and scanning speed were fixed at 2°/min. X-ray fluorescence analyzer (Rigaku ZSX primus, Tokyo, Japan) was used to analyze the composition of geopolymer samples. For this measurement, 10 mm diameter pellets were made using aluminum ring and a high pressing machine. To investigate the chemical bonding inside the geopolymer, the geopolymer powder after crushing was mixed with KBr at a concentration of 1.0–5.0 wt% after which a disk of the mixture was formed by a high press machine. FTIR (FT-IR-4100, JASCO, Tokyo, Japan) spectra were obtained with 20 scans from 400 to 4000 cm^−1^ at a resolution of 2.0 cm^−1^ in transparent mode. SEM (Desktop Scanning Electron Microscope, Hitachi TM3030 Plus, Tokyo, Japan) was used to see the microstructure in geopolymer. For this analysis, all the geopolymer samples were coated with a thin layer of gold on their surfaces after drying with a vacuum pump for 24 h. The compressive strength of geopolymer samples were measured with a testing machine (UH-F50A, SHIMAZU, Kyoto, Japan) and the ratio of compression were fixed to 0.5 mm/min.

## 3. Results and Discussion

### 3.1. US Soil Washing in the Presence of Acid or Base

**Figure 3:**
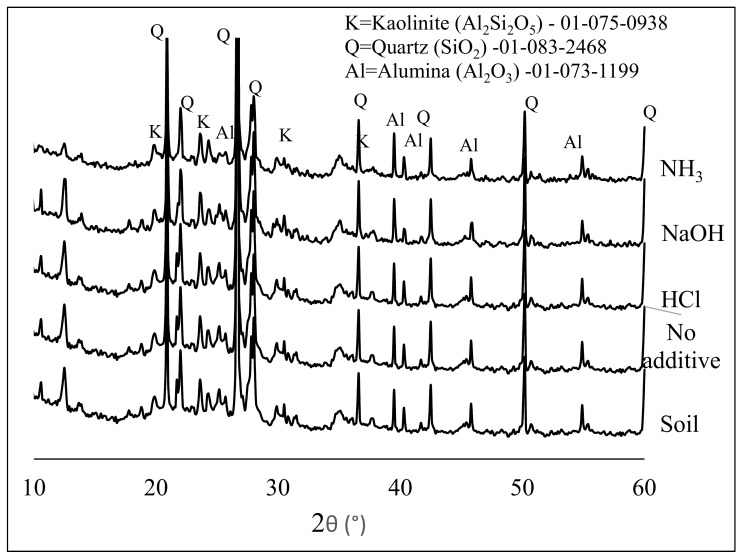
The X-ray Diffractometer (XRD) of soil before and after US treatment.

After US soil washing, the soil obtained after filter-press was dried and analyzed by XRD to determine the effect of sonication on the soil components. Figure 3 shows the XRD diffractograms of the US treated soil with different additive exposed to 1200 W US.

**Figure 4:**
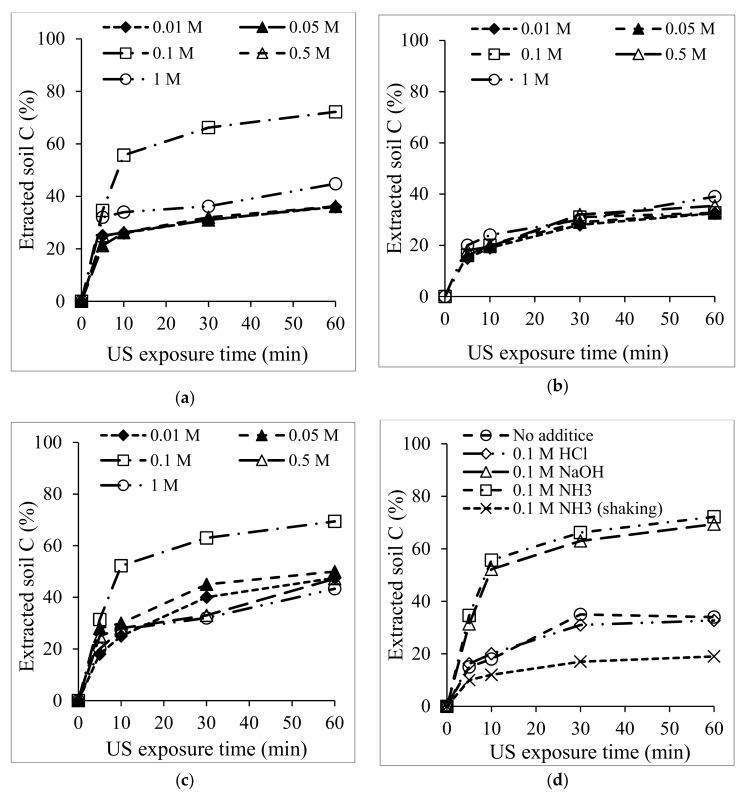
Calculated percentage of carbon extracted under 1200 W US exposure with 0.01–1 M concentration of (**a**) NH_3_, (**b**) HCl, (**c**) NaOH, and (**d**) shows a comparison of the US with no additive and 0.1 M concentration of various additives.

The X-ray powder diffraction patterns of the soils treated with HCl, NaOH, and NH_3_ were compared with that of non-treated soil. The particle mineralogy of the XRD pattern was compared, but no significant difference was observed between the samples. All the diffractograms consisted of quartz (Q), kaolinite (K), and alumina (Al), dominated by quartz peaks around 21 and 27° θ. When the soil was treated with additives, especially when NH_3_ was used, the peak intensity at 25° θ decreased. This indicated the dissolution of alumina. Little or no change was observed in the XRD data before and after treatment. Therefore, XRD analyses are not sufficient for carbon extraction determination. However, when the mineralogical content of the soil was analyzed by XRF, the results showed that US influenced the extraction of about 22% of soil carbon and altered the concentrations of silica and alumina, as shown in Table 1. The concentration of carbon extracted was deduced from the XRF data when different concentrations (0.01–1 M) of the chemical additives (HCl, NH_3_, and NaOH) were used for treatment. The result obtained was plotted as shown in Figure 4, and it was noticed that US treatment for carbon extraction (%) depended on the concentration of the washing solvent and the US washing time. In the case of NH_3_ (e.g., in Figure 4a, when 0.01–1 M NH_3_ was used), 72.2% of the carbon content was extracted with a 0.1 M concentration after 60 min of US exposure. The other concentrations of 0.01 M and 1 M of NH_3_ were not as effective.

HCl, on its part, had no influence regardless of the concentration, even after 60 min US exposure, as seen in Figure 4b. As another base, the effect of 0.001–1 M NaOH was investigated, as shown in Figure 4c. Here, the carbon extraction trend was very similar to that of NH_3_. In total, 69.4% of the carbon content was extracted when 0.1 M NaOH was used, which was slightly less than when NH_3_ was used. Overall, the NH_3_ and NaOH results proved the effectiveness of base additives for carbon extraction from soil under US at 1200 W. The possible reason more carbon was extracted when 0.1 M NH_3_ additives were used is that a weaker base tends to suspend organic matter [20]. 

**Figure 5:**
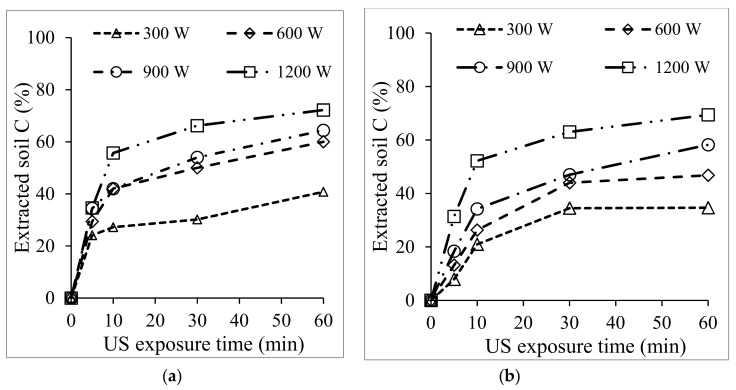
Calculated percentage of organic carbon extracted after 1200 W US exposure at different US power for (**a**) NH_3_ and (**b**) NaOH additives.

It was noted that the amount of carbon extracted with 0.1 M HCl was similar to the amount of carbon extracted when no additive was used for treatment. Interestingly, when the 0.1 M concentration of NH_3_ was used without US, only 19% of the carbon was extracted. This means that the carbon extraction was influenced by US irradiation and not entirely by the additive effect. To confirm this, different US conditions were considered. A plot of the effect of US power from 300–1200 W is shown in Figure 5 below. 

**Figure 6:**
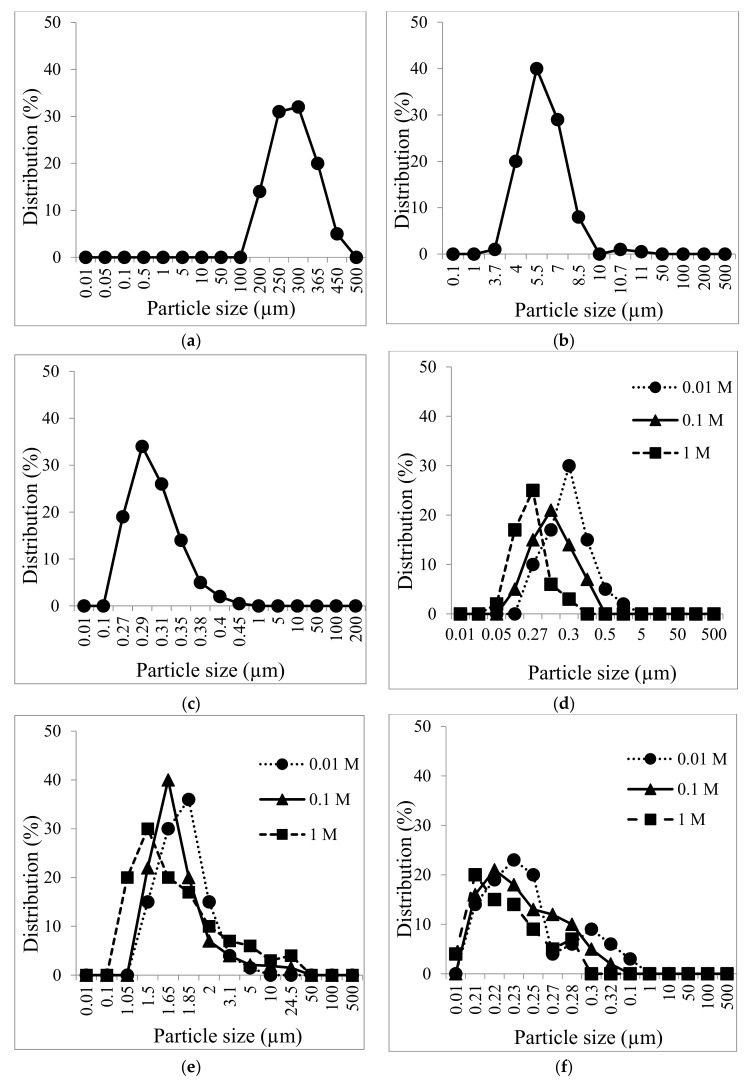
Particle size distribution analyses of soil sample exposed with no additive at (**a**) 0 W (**b**) 600 W and (**c**) 1200 W and with (0.01–1 M) additive under 1200 W with (**d**) NH_3_ (**e**) HCl(**f**) NaOH.

With both solvents, the amount of carbon extracted increased with the US exposure time. In Figure 5a, a slightly steady increase is seen under all the US conditions. But when NaOH was used, saturation was seen after 30 min of US exposure under 300 and 600 W. It is apparent that the amount of extracted C (%) was proportional to the intensity when US was exposed for 60 min. For a better understanding of the effect of US in relation to carbon extraction, the soil fraction was analyzed using a particle size analyzer after exposure to various US conditions. As is known, dispersion by reducing the sample particles is essential during US treatment [19,24], and Figure 6a–f shows us the particle size distribution obtained after exposure. The particle distribution shifted toward 5.5 μm and 0.29 μm for 600 W and 1200 W, respectively. The particle size decreased more when basic additives were used for treatments (d) and (f).

**Figure 7:**
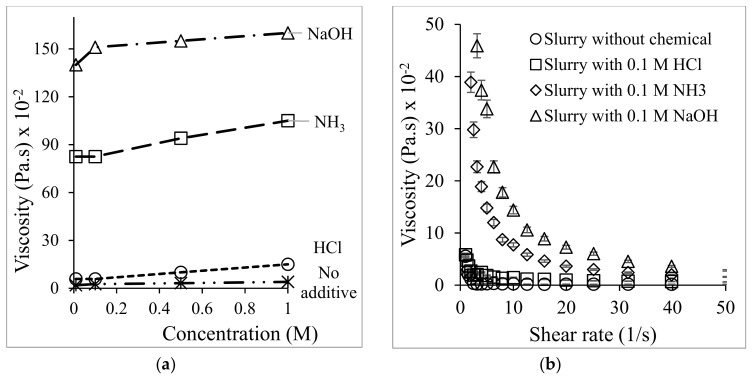
Viscosity changes for 60 min treatment soil slurries with additives (**a**) with different additive concentrations and (**b**) shear rate of 0.1 M additive concentration.

After US treatment, the slurry was observed to be more viscous than it was before exposure, which could be linked to particle breakage. The apparent viscosity of the slurry was measured at a 1s-1 shear rate as a function of the additive concentration in Figure 7a and a different shear rate in Figure 7b with a 0.1 M concentration of each additive. As seen in Figure 7b, the viscosity decreases when the shear rate increases from 1 to 1000 s^−1^, suggesting that the samples followed a pseudoplastic flow behavior. The viscosity curve of NH_3_- and NaOH-treated soils showed a thixotropic shear rate, especially in the NaOH cases. This might be because strong alkalis, such as NaOH, produce metal colloids for Si(OH)_4_ or Al(OH)_3_, meaning that the hydrogen interaction influenced the viscosity and the thixotropic behavior [37] in the base cases. 

During this study, 0.1 M NH_3_ was considered the most effective surfactant for treatment. So far, no report exists emphasizing the adverse effects of NH_3_ on health at concentration levels found in the natural environment. Shinma et al. investigated the mechanisms between soil and alkali interactions [40]. It was seen that at higher concentrations, clay alkali interactions produced new compounds and also affected the clay structure, which led to soil swelling [41,42].

### 3.2. Fabrication and Properties of Soil Geopolymers

**Figure 8:**
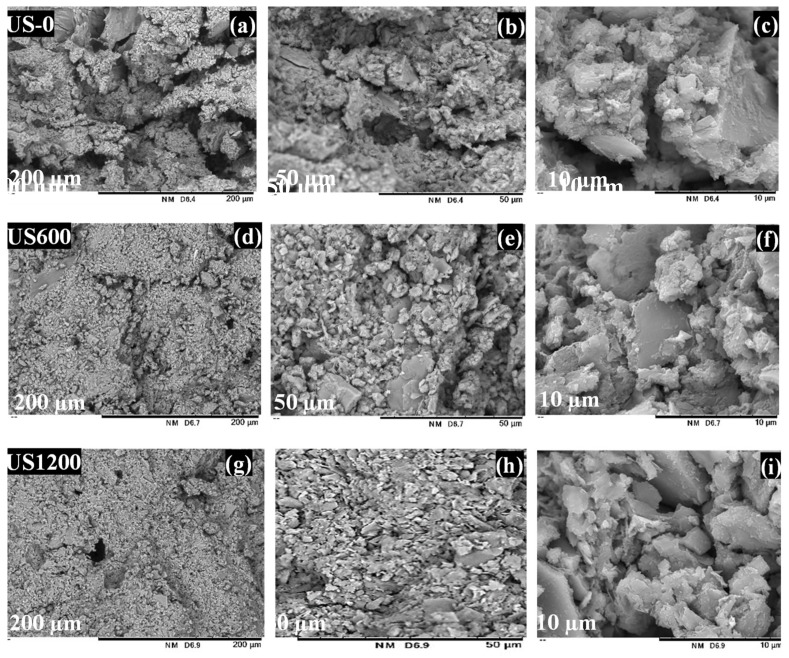
Microstructure of geopolymer sample after before and after ultrasonic treatment at different intensities. (0 W, 600 W, and 1200 W) of NH3, (**a**–**c**) US-0, (**d**–**f**) US-600, (**g**–**i**) US-1200.

Before US treatment, from Table 1 we can see that the original soil used for the experiment had 3.6% organic carbon content, 50.4%, 18.8% of SiO_2_ and AL_2_O_3_ contents, respectively. However, after US treatment, NaOH like NH_3_ surfactant extracted 1/3 the carbon content, while an increase in SiO_2_ from 65.2%–63.3% was noticed as Al_2_O_3_ contents decreased from 16.2%–16.1%. For geopolymer, three samples were considered: the original soil without US treatment (US-0), soil washed with 0.1 M NH_3_ additive under 600 W US (US600), and soil washed with 0.1 M NH_3_ additive under 1200 W US (US1200). When the soils powders were mixed with the alkaline activator, the workability of the paste was best with the soil which was treated under 1200 W(US1200). After forming the geopolymer matrix, the morphology of the geopolymers was analyzed and the SEM images are shown in Figure 8. The geopolymer formed from untreated soil in Figure 8a shows a clear boundary between the particles and the alkalis activator in the geopolymers. This result explains that the layer has a less compact structure which can clearly be seen with high focus of 10 µm in Figure 8c. However, in the case of US1200 for Figure 8g–i, it can be seen that the effect of the high power US-led to fine soil powder. The geopolymer interface shows a more discrete matrix indicated by the globular unit and a few unreacted particles (layered structure). This means that the soil particles and the alkaline activator in the geopolymers formed dense matrix with fewer gaps at the interfaces, signifying that US was effective in the soil slurry dispersion in the aqueous medium.

**Figure 9:**
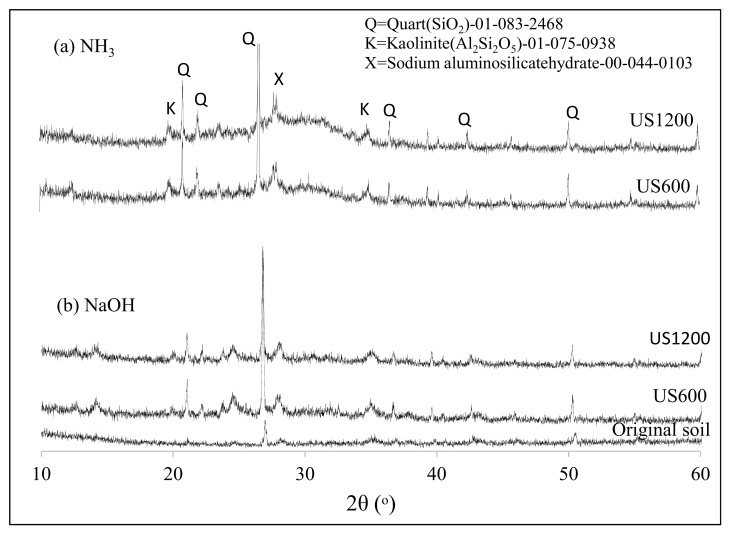
XRD patterns of original soil and soil after geopolymerization under different ultrasound power, (**a**) NH3, (**b**) NaOH.

The XRD of the geopolymers formed from NH_3_ above was analyzed and just for clarity it was compared with geopolymer formed from soil treated with NaOH and soil treated with no additive. The geopolymers consisted mainly of amorphous alumino-silicate products with similar amount or slight increase of SiO_2_ and Al_2_Si_2_O_5_ crystals relative to original sample as shown in Figure 9. The synthesized geopolymers diffractogram showed the presence of residual peaks relating to SiO_2_ (21.11° and 26.8°). One major difference between the XRD patterns of the geopolymer from NaOH treated soil and NH_3_ soil was the occurrence of a broad diffuse hump between 25° and 35° 2θ in Figure 9a when the soil was washed with NH_3_. This amorphous peak corresponded to the aluminosilicates that were formed at the binder phase in the geopolymer matrix. The component analyses of the geopolymer in Table 2 show increases in the SiO_2_ component and in the Al_2_O_3_ component, which are apparent for NH_3_.

**Figure 10:**
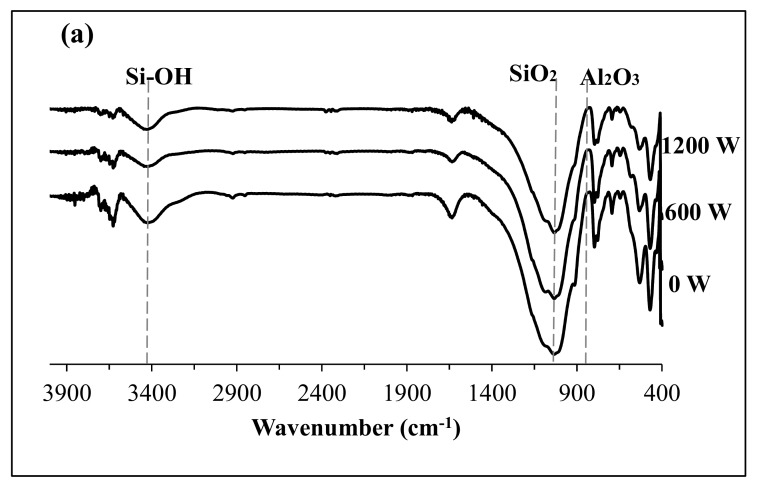

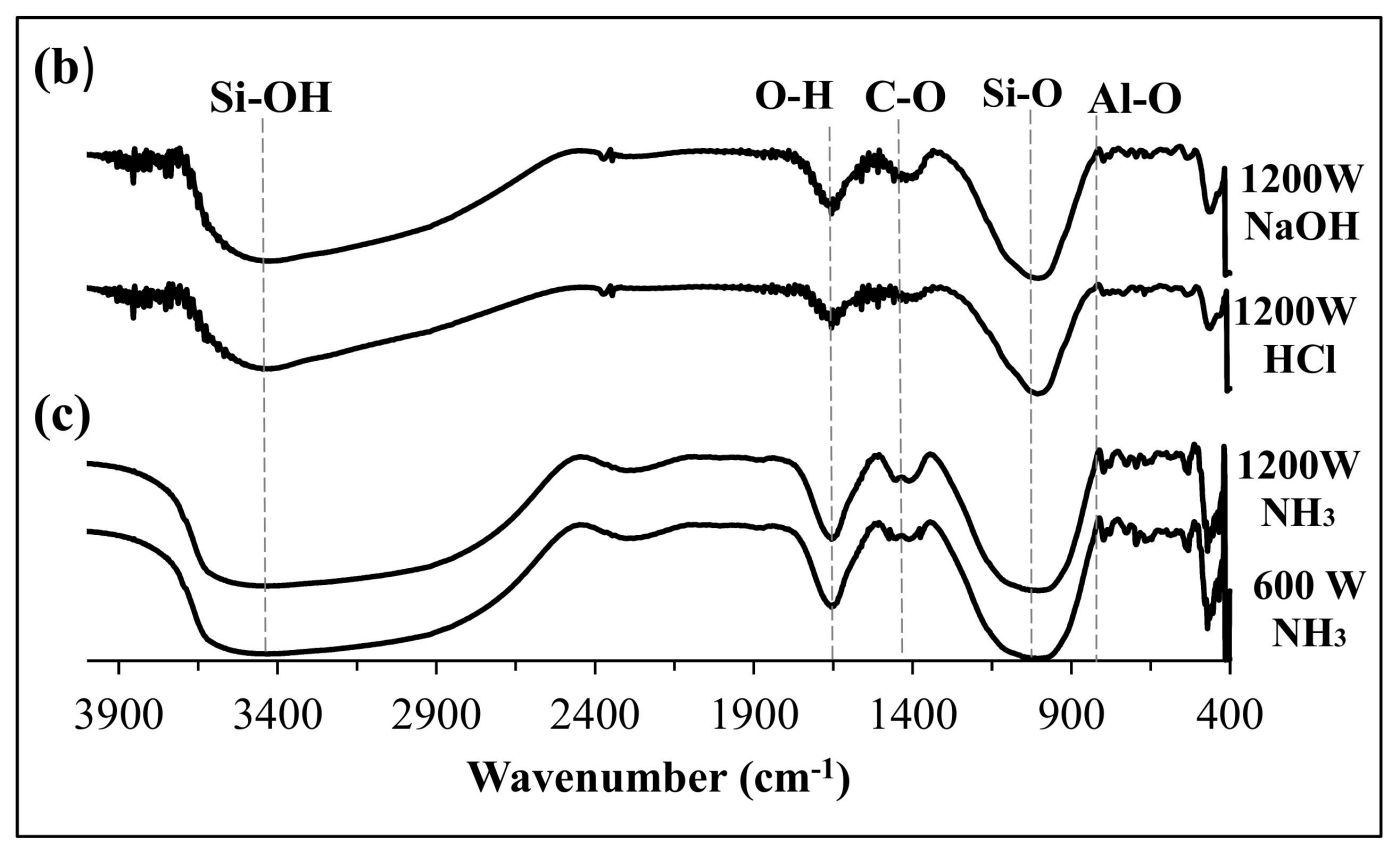
FT-IR analysis after geopolymerization of 1200 W US (**a**) treated soil with no additive. (**b**) treated with 0.1 M NaOH (top) and 0.1 M HCl (bottom) ) and (**c**) treated with 0.1 M NH3.

To confirm the formation of geopolymer alumino-silicate, FT-IR spectra for the US treated soil and geopolymer samples were analyzed as shown in Figure 10. The FT-IR data of the additives were seen to have numerous transmission bands around 900 cm^−1^, 1100 cm^−1^ and 3200–3500 cm^−1^ for Al–O, Si–O and Si–OH groups, respectively [36]. In Figure 10a, 0 W indicates no US treatment, while 600 W and 1200 W indicate their respective US powers without additive.

From the FT-IR, the main feature of the spectra of the US treated powder is the appearance of the central band between 1107 and 1040 cm^−1^ characterized by the asymmetric stretching of Si–O–Si. The characteristic stretching vibration of Al–O was observed in the geopolymers formed from non-additive treated soil as shown in Figure 10a. In Figure 10b, the Si–O stretching band at about 1100 cm^−1^ of the geopolymer formed from treated soil slightly shifted towards the lower wavenumber region, indicating that condensation occurred in the soil between SiO_2_ and the sodium silicate. At 1490–1650 cm^−1^, the characteristic carbonate band appeared. This band from sodium carbonate was formed during the absorption of CO_2_ gas from the atmosphere by the sodium silicate. 

**Figure 11:**
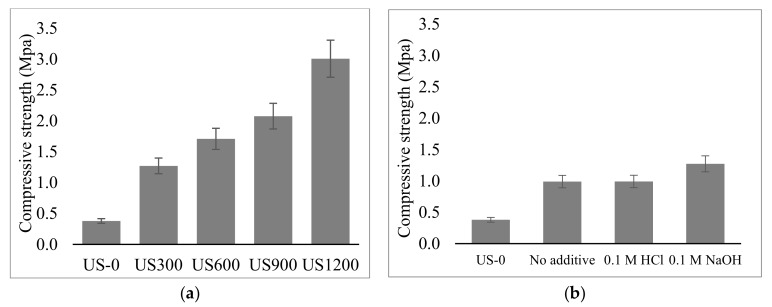
Compressive strength of (**a**) geopolymer from 0.1 M NH_3_ US treated soil at different power. (**b**) geopolymer from the US treated soil with different additive.

Since strength is one of the major characteristics of a geopolymer, the mechanical strength of the geopolymer matrix was analyzed and plotted in Figure 11. Four standard-cured strength specimens were made from the same sample and tested at the same age. Two plots were made, the compressive strength of the geopolymer formed from soil which was washed with 0.1M NH_3_ additive under different US power (0–1200 W) (Figure 11a), and the geopolymer made from soil washed with 0.1 HCl and NaOH (Figure 11b) as shown below.

In Figure 11a, 0.1 M NH_3_, an increase in US power led to an increase in the compressive strength, from 0.4 MPa to 3.0 MPa when 1200 W US (US1200) treated soil was used. This result was mainly related to the morphological structure of the geopolymer, as a higher US power of 1200 W created more fine particles which required less geopolymerization reaction time as compared to larger soil particles in the case of lower powers [43]. When similar US conditions of 1200 W were applied to soils that were treated with other additives like HCl and NaOH, although their particle size was assumed to be the same, their compressive strengths were far lesser than that of NH_3_ mentioned earlier. The main reason for this could be attributed to the carbon content in the material [15]. The particle size influenced the strength when no additive, as well as when 0.1 M HCl and 0.1 M NaOH was used as shown in Figure 11b. 

During geopolymerization, to ensure sufficient reaction multiples of 7 days (7, 14 or 28) are commonly considered for maturity testing in civil engineering at room temp (25–30 °C) [44]. This is because, during geopolymerization, the strength of kaolin geopolymers increases with aging time as the dissolution increases with aging days [45]. As a result geopolymers would only achieve complete geopolymerization after a particular time frame. In this study, although the compressive strength of the prepared geopolymers was considered high with respect to the compressive strength when the other surfactants were used for treatment, on the other hand it was not as high as most reported geopolymers. However, the geopolymer was within the accepted range for geopolymers compressive strength tested after 1 day curing. Reports when similar specimens were cured at 80 °C also showed similar strength after 1 day of curing [46,47]. 

## 4. Conclusions

Soil slurry, which was considered as waste, was recycled to prepare geopolymers after having been treated with high power ultrasound (US). Due to the limitation of land fields for waste soil disposal, soil decarbonation is said to be a necessity for soil recycling. Under 28 kHz US exposure, 60 min was sufficient to extract 72.2% of organic carbon from excavated soil when 0.1 M NH_3_ solution was used as washing solvent. Geopolymer formed from the resultant soil showed a remarkable compressive strength of 3 MPa after curing at 80 °C for 24 hr due to the low proportion of carbon content after treatment. Although the compressive strength acquired with 0.1 M NH_3_, is thought to be negligible it is significant when compared to geopolymers formed with soils treated with similar concentration of HCl and NaOH. The compressive strength is not as high when compared to the other reported geopolymers but it is within the accepted range for geopolymers after 1 day curing. Since the compressive strength depends on the particle size and carbon content in the soil, more research is required to develop a geopolymer with better strength by altering the concentration of alkalis activator and other ultrasonic parameters.

## Figures and Tables

**Table 1:** Chemical composition (mass %) of raw sample (soil), before treatment and after 1200 W US (ultrasound) treatment with different concentrations of additive.

				HCl			NH_3_			NaOH	
US Treatment	Soil	No additive	0.01 M	0.1 M	1 M	0.01 M	0.1 M	1 M	0.01 M	0.1 M	1 M
C	3.6	2.4	2.4	2.4	2.1	2.3	1.0	1.9	1.9	1.1	2.0
Na_2_O	1.3	1.4	2.0	1.84	2.2	2.1	2.1	2.0	2.2	2.8	5.0
SiO_2_	50.4	55.3	63.6	62.8	63.9	64.8	65.2	63.5	64.2	63.3	62.6
Al_2_O_3_	18.8	17.1	15.8	15.4	14.7	15.7	16.2	16	16	16.1	15.3
Fe_2_O_3_	4.1	4.0	3.9	3.2	2.9	3.9	4.1	4.2	4.1	3.9	3.4

**Table 2:** XRF of geopolymers.

		Mass %	
	No Additive	NH_3_	NaOH
CO_2_	21.5	11.6	12.4
Na_2_O	20.9	17.1	18
Al_2_O_3_	11	13.9	12.7
SiO_2_	40.4	48.9	48.8
